# Chitosan as a Coagulant to Remove Cyanobacteria Can Cause Microcystin Release

**DOI:** 10.3390/toxins12110711

**Published:** 2020-11-10

**Authors:** Maíra Mucci, Iame A. Guedes, Elisabeth J. Faassen, Miquel Lürling

**Affiliations:** 1 Aquatic Ecology and Water Quality Management Group, Department of Environmental Sciences, Wageningen University, Droevendaalsesteeg 3a, 6708 PB Wageningen, The Netherlands; els.faassen@wur.nl (E.J.F.); miquel.lurling@wur.nl (M.L.); 2 Laboratory of Microbiology, Wageningen University, Stippeneng 4, 6708 WE Wageningen, The Netherlands; iame.alvesguedes@wur.nl; 3 Wageningen Food Safety Research, Wageningen Research, Akkermaalsbos 2, 6708 WB Wageningen, The Netherlands

**Keywords:** lake restoration, cyanobacteria bloom control, membrane integrity, *Microcystis aeruginosa*, microcystin

## Abstract

Chitosan has been tested as a coagulant to remove cyanobacterial nuisance. While its coagulation efficiency is well studied, little is known about its effect on the viability of the cyanobacterial cells. This study aimed to test eight strains of the most frequent bloom-forming cyanobacterium, *Microcystis aeruginosa*, exposed to a realistic concentration range of chitosan used in lake restoration management (0 to 8 mg chitosan L^−1^). We found that after 1 h of contact with chitosan, in seven of the eight strains tested, photosystem II efficiency was decreased, and after 24 h, all the strains tested were affected. EC_50_ values varied from 0.47 to > 8 mg chitosan L^-1^ between the strains, which might be related to the amount of extracellular polymeric substances. Nucleic acid staining (Sytox-Green^®^) illustrated the loss of membrane integrity in all the strains tested, and subsequent leakage of pigments was observed, as well as the release of intracellular microcystin. Our results indicate that strain variability hampers generalization about species response to chitosan exposure. Hence, when used as a coagulant to manage cyanobacterial nuisance, chitosan should be first tested on the natural site-specific biota on cyanobacteria removal efficiency, as well as on cell integrity aspects.

## 1. Introduction

Cyanobacteria play an essential role in oxygen production, being responsible for half of the ocean’s primary production [1]. However, cyanobacterial species may form intense blooms under certain conditions, which have severe impacts on water bodies, such as increased water turbidity, nocturnal depletion of oxygen, fish kills, and malodour [2,3]. In addition, cyanobacteria can produce toxins that are harmful to aquatic and terrestrial organisms, including humans and dogs, impeding water bodies use for recreational activities, drinking water production, fishing, and agricultural use and, consequently, causing severe economic losses [4,5,6,7,8]. The main cause of cyanobacterial blooms is the excess of nutrient supply to waterbodies (eutrophication) [9]. Thus, to manage the problem, nutrients must be limited. The classical and most straightforward approach is to reduce the external nutrient input [10,11,12]; however, adequate catchment control is not always feasible for economic reasons [13]. In addition, in cases where the internal loading is the primary nutrient source due to long-term diffuse load (e.g., [14,15]) the reduction in external nutrient sources will be inefficient [16,17]. Hence, to speed-up system recovery and minimize nuisance, in-lake measures have been recognized as a feasible solution [13,18].

In this context, geo-engineering materials, like the use of low doses of flocculants (e.g., PolyAluminium Chloride (PAC) or iron chloride—“Floc”) followed by the addition of natural soils or modified clays (e.g., lanthanum modified bentonite or aluminium modified zeolite—“Lock/Sink”) have gained attention as useful tools to mitigate the effects of eutrophication. This “Floc & Lock/Sink” technique can remove cyanobacteria from the water column while blocking P efflux from the sediment [19]. This approach has been implemented effectively using PAC or iron chloride as coagulant [14,15,20]. Recently, an organic coagulant, chitosan, has gained attention as a possible alternative for inorganic metal-based coagulants [21].

Chitosan is an organic polymer synthesized by alkaline deacetylation of chitin, a biopolymer extracted from shellfish and crustaceans [22]. Chitosan acts as a cationic polyelectrolyte when protonated in an acidic medium; thus, its free amino groups interact with the negatively charged cyanobacterial cell wall [22,23,24]. Due to its long polymer chain, chitosan can also attach to the cells, forming bridges that entrap the cells [22]. Chitosan is frequently viewed as an eco-friendly and non-toxic coagulant [21,24,25,26], and besides its coagulation property, chitosan is also known for its antimicrobial activities [27,28,29,30,31], and it has been even used to preserve food [32,33,34].

Several studies have used chitosan to remove cyanobacteria or dinoflagellates from the water column, some using chitosan-Modified Local Soils/Sand (MLS, e.g., [21,25,35,36,37,38,39]), others adding first only chitosan, followed by soils/clays (e.g., [40,41,42,43]) and recently a chitosan fiber has been used [44]. However, in only a few of these studies, the possible chitosan effects on the algal cells viability were investigated. From these studies, some did not find any adverse effect on the cyanobacterium *Microcystis aeruginosa* (e.g., [40,41,42,45]), whereas others showed a detrimental effect on the cyanobacterium *Cylindrospermopsis raciborskii* and growth inhibition in the dinoflagellate *Amphidinium carterae* [25,41]. A more recent study from our group indicated rapid cell lysis of some cyanobacterial species when incubated with chitosan, but a less severe impact on *M. aeruginosa* [46], and in this study, cyanotoxin release was not analyzed. In fact, only a few studies so far have addressed cyanotoxin release caused by chitosan; in some of these studies, toxins were released, and in others, toxins were not released [35,41,47,48,49].

Our present study aims to extend the knowledge of possible side effects caused by chitosan on the cyanobacterium, *Microcystis aeruginosa*. Possible materials to manage blooms must be efficient, easy to apply, cheap, and safe [50]. Therefore, an environmentally safe management strategy should be selected, and methods that cause cell damage and toxin release must be applied carefully or should be avoided [51].

We tested the response of the most frequently encountered bloom-forming cyanobacterium, *M. aeruginosa* [52,53,54], to a realistic concentration range of chitosan as used in lake restoration management [49]. Since intraspecific variation was observed in other species [46], we tested eight different strains of *M. aeruginosa*. The effect of chitosan was evaluated by analysing the photosystem II efficiency and filterable Chlorophyll-*a* concentration. Besides, we analysed cell membrane integrity and the extracellular microcystin concentration. We hypothesized (1) that chitosan would negatively affect all the *M. aeruginosa* strains tested only at the highest chitosan dose, (2) that sensitivity to chitosan will not differ between strains, and (3) that cell lysis followed by toxin release will be observed only at the highest chitosan dose.

## 2. Results

**Figure 1:**
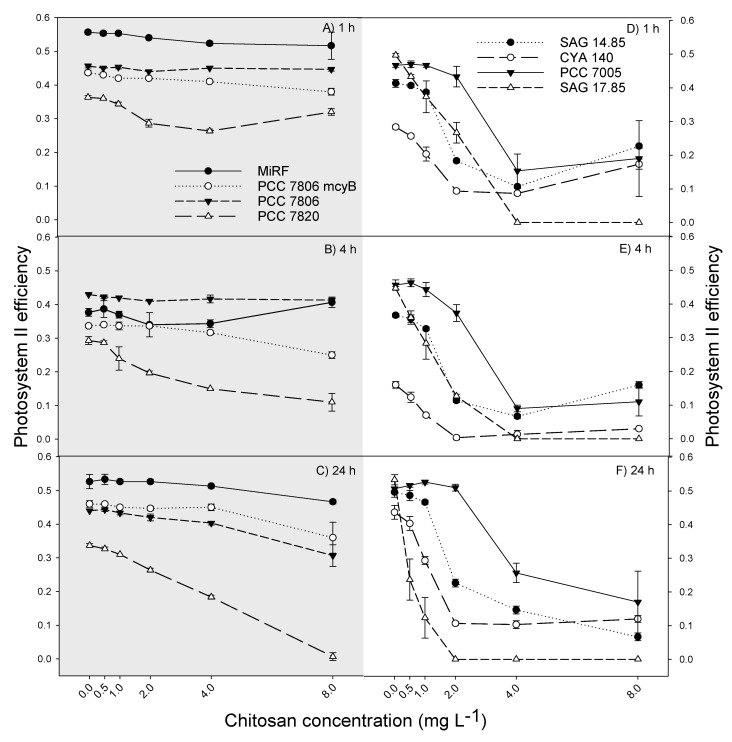
(**A**,**D**) Photosystem II efficiency (PSII) for all 8 strains tested after 1 h, (**B**,**E**) 4 h, and (**C**,**F**) 24 h exposure to different concentrations of chitosan (0 to 8 mg L^−1^). Error bars indicate standard deviation (*n* = 3). Grey graphs on the left show the strains with slow response (MiRF-1, PCC 7806 ΔmcyB-, PCC7806, and PCC 7820), and graphs on the right show strains with an earlier response (SAG 1485, CYA 140, PCC 7005 and SAG 1785).

The eight *M. aeruginosa* strains tested were affected differently by chitosan. Considering the effect of chitosan on the Photosystem II (PSII) efficiency, we could divide the *M. aeruginosa* strains based on their response into two groups: (1) the strains that have a delayed response to chitosan (MiRF-1, PCC 7806 ΔmcyB, PCC 7806 and PCC 7820) and (2) the more sensitive strains with an earlier response (SAG 14.85, CYA 140, PCC 7005 and SAG 17.85) (Figure 1). After 1 h of contact with chitosan, hardly any effect on the PSII efficiency from the first group of strains was observed (Figure 1A; Table A1). After 4 h, the effects on PSII efficiency became visible at the highest concentration for the strain PCC 7806 ΔmcyB and from 1 mg L^−1^ for the strain PCC 7820 (Figure 1B), while after 24 h, these effects became more pronounced (Figure 1C), and the PSII efficiencies in strains PCC 7806 and MiRF-1 were reduced at 8 mg chitosan L^−1^ (Figure 1C).

The response of strains from the second group was different; SAG 14.85, CYA 140, PCC 7005, and SAG 17.85 showed already after 1 h a sigmoidal decrease in PSII efficiency with higher chitosan concentrations (Figure 1D). This pattern persisted after 4 and 24 h of chitosan incubation (Figure 1E,F).

**Figure 2:**
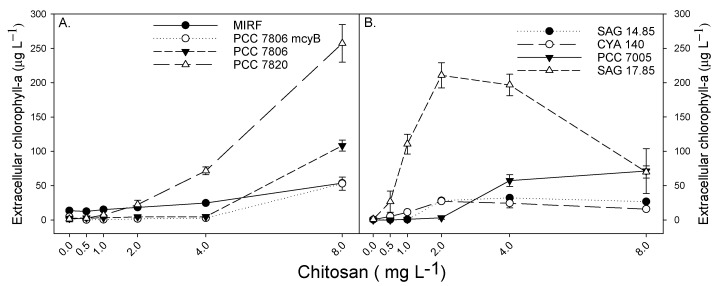
Extracellular Chlorophyll-*a* concentration for MiRF, PCC 7806 ΔmcyB, PCC 7806 and PCC 7820 (**A**) and for SAG 14.85, CYA 140, PCC 7005 and SAG 17.85 (**B**) after 24 h exposure to different concentrations of chitosan (0 to 8 mg L^−1^). Error bars indicate standard deviation (*n* = 3).

**Figure A1:**
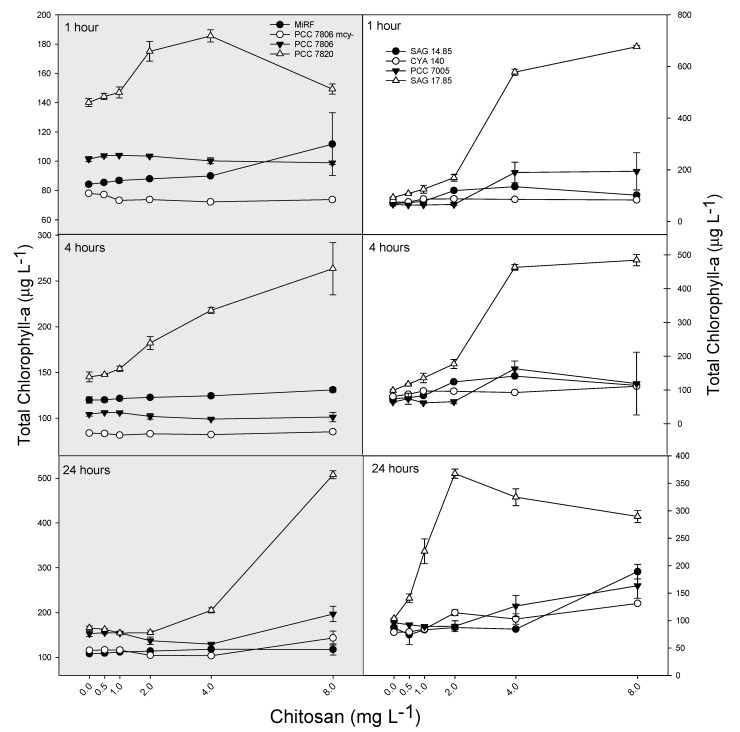
Total Chlorophyll-*a* concentration for all 8 strains tested after 1 h, 4 h and 24 h exposure to different concentrations of chitosan (0 to 8 mg L^−1^). Error bars indicate standard deviation (*n* = 3). Graphs on the left show strains with a slow response (MiRF-1, PCC 7806 ΔmcyB, PCC7806, and PCC 7820) and graphs on the right show strains with an earlier response (SAG 14.85, CYA 140, PCC 7005 and SAG 17.85).

**Figure A2:**
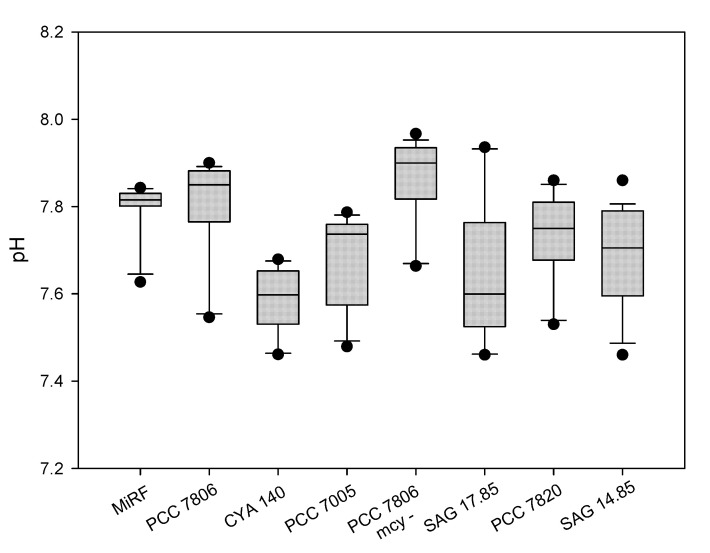
Box plot showing median pH between the treatment, 10th, 25th, 75th and 90th percentiles with error bars (*n* = 18) for each strain.

**Figure A3:**
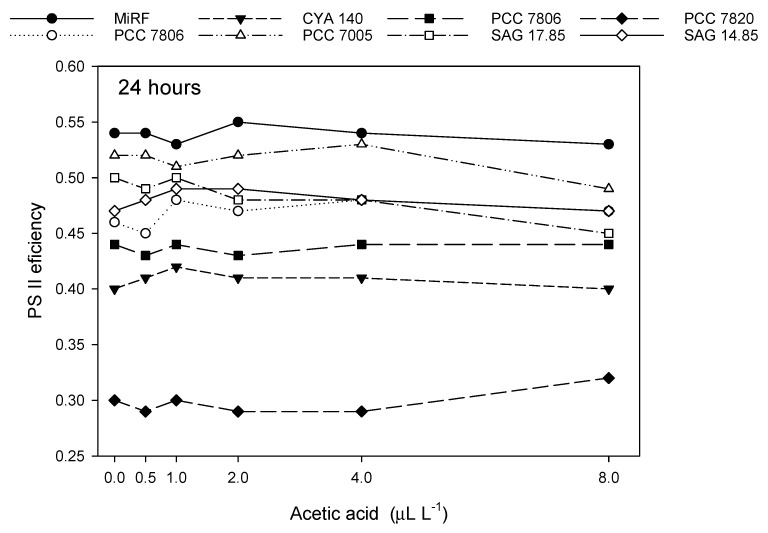
PSII efficiency of all the eight strains tested after 24 h exposure to different acetic acid concentration, the same used for the chitosan treatments.

The strains PCC 7820 and SAG 17.85 showed an increase in total and extracellular Chlorophyll-*a* concentrations as a response to the chitosan treatments (Figure 2 and Figure A1). At the end of the experiment, the total Chlorophyll-*a* concentration of both strains, when exposed to 8 mg chitosan L^−1^, was three times higher than in the control. The other six strains used (MiRF-1, PCC 7806 ΔmcyB, PCC 7806, SAG 14.85, PCC 7005, and CYA 140) only increased total Chlorophyll-*a* compared to control after 24 h and in the highest chitosan concentration used. In all strains, pH variation between treatments remained low and below 0.5 units (Figure A2). The addition of acetic acid did not affect PSII efficiency (Figure A3).

In the first group of strains (MiRF-1, PCC 7806 ΔmcyB, PCC 7806, and PCC 7820), extracellular Chlorophyll-*a* concentrations were elevated at the highest chitosan dose (Figure 2A). On the contrary, in the second group, at lower chitosan doses, elevated extracellular Chlorophyll-*a* concentrations were observed (Figure 2B). Extracellular Chlorophyll-*a* concentrations differed considerably among strains, with the highest concentration found in strain PCC 7820 (257 µg extracellular Chlorophyll-*a* L^−1^) and the lowest in strain CYA 140 (16 µg extracellular Chlorophyll-*a* L^−1^), both at the 8 mg chitosan L^−1^ treatment (Figure 2).

EC_50_ values for MiRF-1, PCC 7806 ΔmcyB, and PCC 7806 could not be calculated because the values exceeded the highest dose used (8 mg L^−1^) (Table 1). SAG 17.85 was the most sensitive strain with the lowest EC_50_ value (0.47 mg chitosan L^−1^) followed by CYA 140 (1.06 mg chitosan L^−1^), SAG 14.85 (1.71), PCC 7005 (3.44) and PCC 7820 (4.51, Table 1). One-way ANOVA showed a difference between the strains (F_4,10 =_ 47.74; *p* < 0.001) and the Tukey post-hoc test divided the strains into three different groups: 1) SAG 17.85, CYA 140 and SAG 14.85 were the most sensitive, 2) followed by PCC 7005 and 3) PCC 7820 (Table A1). MiRF-1, PCC 7806 ΔmcyB, and PCC 7806 were the least sensitive.

**Figure 3:**
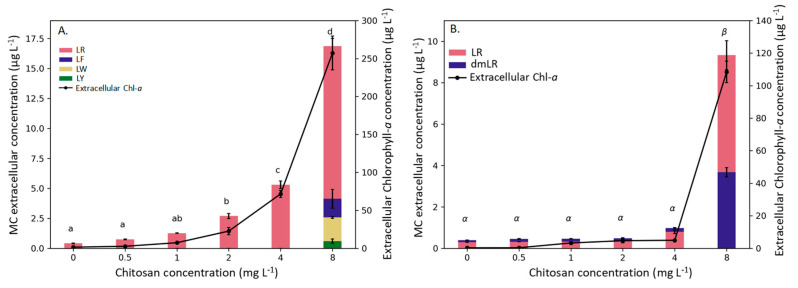
Extracellular MCs (bars) and extracellular Chlorophyll-*a* (line) after 24 h of chitosan exposure for PCC 7820 (**A**) and PCC 7806 (**B**). Errors bars indicate standard deviation (*n* = 3). Letters represent a statistical difference (Tukey pairwise comparisons *p* < 0.05).

**Figure A4:**
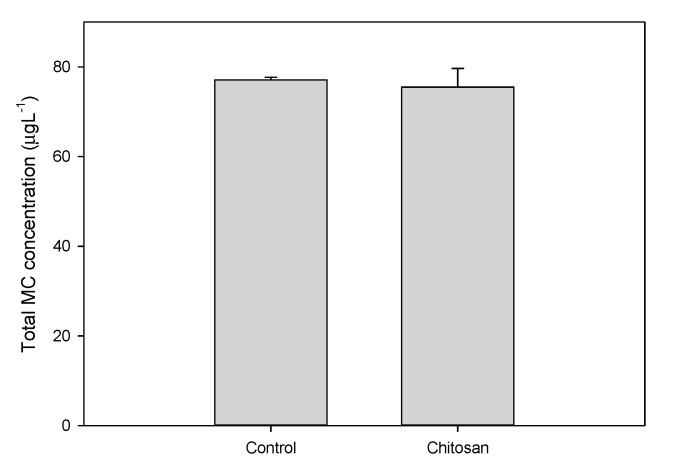
Total MC incubated for 24 h with only WC medium (Control) and with WC medium plus 8 mg chitosan L^−1^ (Chitosan).

Extracellular microcystins (MCs) concentrations were below the detection level in the filtrates from incubations of MiRF-1 exposed to 0 to 4 mg chitosan L^−1^, while the variant MC-LR was detected at 8 mg chitosan L^−1^, but below the level of quantification. Likewise, in strain CYA 140, no extracellular MCs were detected in incubations exposed to 0 to 1 mg chitosan L^−1^, whereas MC-LR was detected, yet not quantifiable, at 2, 4, and 8 mg chitosan L^−1^. On the other hand, in the strain PCC 7820, extracellular MCs increased with an increasing chitosan concentration (one-way ANOVA; F_5,11_ = 4516.5; *p* < 0.001), and already at 1 mg chitosan L^−1,^ the extracellular MC concentration was significantly higher than in the control (Figure 3A). In the strain PCC 7806, the extracellular MC variants LR and dmLR increased only at the highest chitosan dose (*Kruskal–Wallis* One Way Analysis of Variance on Rank; s H_5_ = 16.251; *p* = 0.006) (Figure 3B). The MC analysis was not affected by the presence of 8 mg of chitosan L^−1^, as demonstrated in an incubation experiment (Student’s *t*-test, *p* = 0.552, Figure A4).

**Figure 4:**
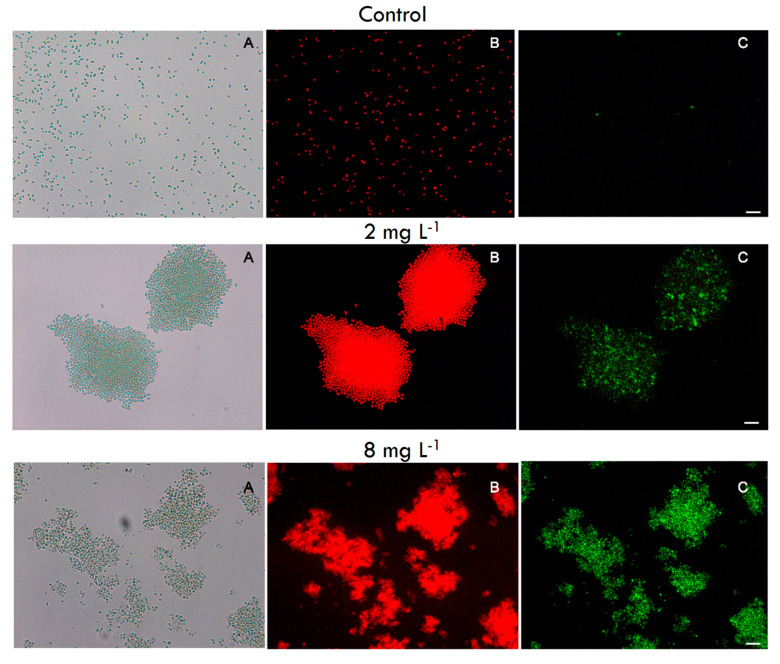
Fluorescence images of PCC 7820 cells in the control (upper pictures **A**–**C**), 2 mg chitosan L^−1^ (middle pictures **A**–**C**), and 8 mg chitosan L^−1^ (lower pictures **A**–**C**). The panels A shows bright-field images, B the cyanobacteria autofluorescence (red), and C the intracellular accumulation of Sytox^®^ Green (green). Scale bar: 20 µm.

**Figure A5:**
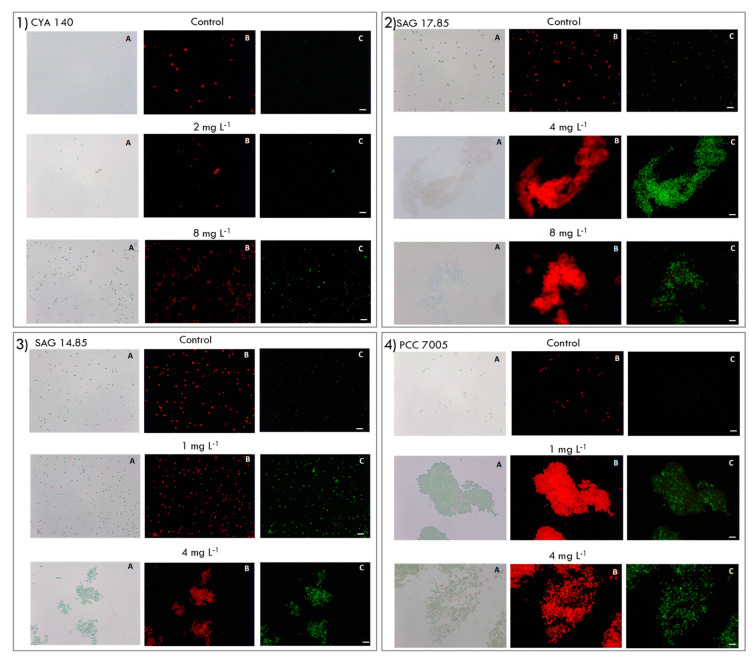
Four panels showing the fluorescence images of four strains (1: CYA 140, 2: SAG 17.85, 3: SAG 14.85 and 4: PCC 7005. For each panel the cells in the control are the upper pictures (**A**–**C**), 2, 1 or 4 mg chitosan L^−1^ are the middle pictures (**A**–**C**), and 8 or 4 mg chitosan L^−1^ are the lower pictures (**A**–**C**). For all the panels, picture A shows bright-field images, B the cyanobacteria autofluorescence (red), and C the intracellular accumulation of Sytox^®^ Green (green). Scale bar: 20 µm.

**Figure A6:**
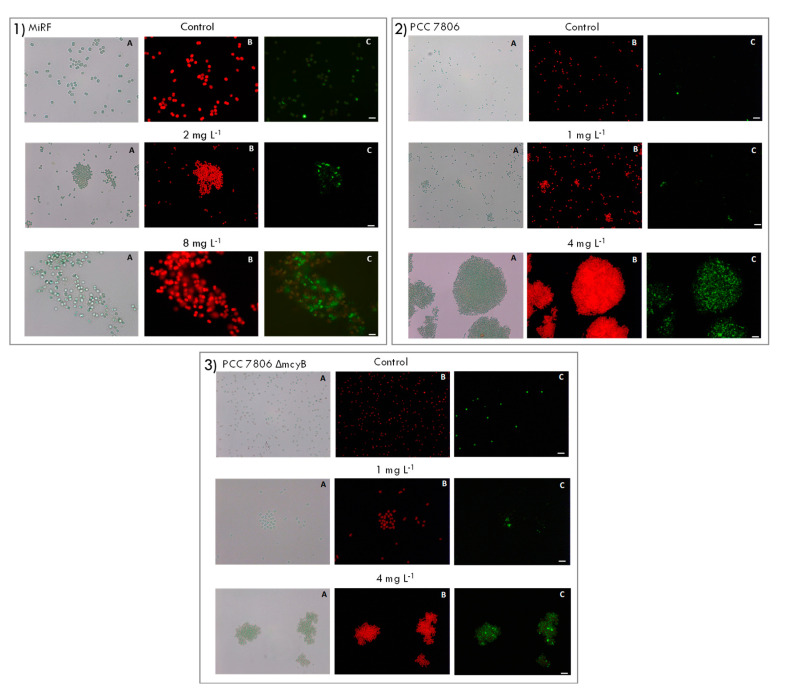
Three panels showing the fluorescence images of three strains (1: MiRF, 2: PCC 7806, and 3: PCC 7806 ΔmcyB. For each panel, the cells in the control are the upper pictures (**A**–**C**), 2, 1 or 4 mg chitosan L^−1^ are the middle pictures (**A**–**C**), and 8 or 4 mg chitosan L^−1^ are the lower pictures (**A**–**C**). For all the panels, picture A shows bright-field images, B the cyanobacteria autofluorescence (red), and C the intracellular accumulation of Sytox^®^ Green (green). Scale bar: 20 µm.

For the four most sensitive strains (SAG 14.85, PCC 7005, PCC 7820, SAG 17.85), except for CYA 140, the cell membrane permeability test showed differences between cells exposed to chitosan at each of the concentrations used and the non-exposed (control) cells (Figure A5). Most of the strains had similar results as the strain PCC 7820: the non-exposed cells showed only the natural red fluorescence (Figure 4; panel control B) and no intracellular accumulation of Sytox Green (Figure 4; panel control C). However, in the treatment with chitosan, intracellular accumulation of Sytox Green was observed (Figure 4; panel 2 mg L^−1^ C), while the accumulation was even more substantial at the highest chitosan dose (8 mg L^−1^), indicating membrane damage (Figure 4; panel 8 mg L^−1^ C). In the less sensitive strains MiRF-1, PCC 7806 ΔmcyB, and PCC7806, such intracellular accumulation of Sytox Green was only strongly observed at the highest concentration (Figure A6). For certain strains (SAG 14.85, PCC 7005, PCC 7806 and PCC 7806 ΔmcyB), it was not possible to analyze at the highest concentration the cell damage via Sytox because the cells were already destroyed. 

## 3. Discussion

Chitosan has recently received attention as a coagulant to remove cyanobacteria from waterbodies. It has good flocking properties depending on water chemistry [55] and, combined with a ballast, can remove cyanobacteria effectively from the water column [21,25,45]. However, effects on the viability of flocked cyanobacteria have received less attention but are of great importance as chitosan may cause cell membrane damage in bacteria [56] and may cause cyanotoxin release [41]. Our study filled in this research gap by testing the response of eight different *Microcystis* strains to chitosan, while also measuring extracellular MCs.

Our results are not in agreement with the hypothesis that chitosan would affect the *M. aeruginosa* strains only at a high dose. In five strains, an immediate negative impact was detected, while in three less sensitive strains (MiRF-1, PCC 7806, and PCC 7806 ΔmcyB), a significant reduction in PSII efficiency was observed only at the highest chitosan dose tested. PSII is one of the reaction centers responsible for transporting energized electrons to accomplish photosynthesis [57]; thus, a decrease in PSII efficiency reflects damage to the thylakoid membrane and gives insight into the physiological status of the cells. The reduction in PSII efficiency most probably reflects increased membrane permeability and cell lysis that is a result of the cationic amino groups (C-H_3_^+^) of chitosan interacting with negatively charged cyanobacterial cell membranes [56]. A significant reduction in the PSII efficiency of MiRF-1 cells was also observed in our previous work [46]. In contrast, some studies found no decrease in PSII efficiency at similar chitosan concentrations [40,41,42,45]. These studies had incubated lake water infested with cyanobacteria only for one hour, which might be too short to evoke a measurable effect. The strains SAG 14.85, CYA 140, PCC 7005, and SAG 17.85 were strongly affected after 1 h incubation, but in MIRF-1, PCC 7806, PCC 7820, and PCC 7806 ∆mcyB, negative effects became apparent after 24 h incubation. Hence, the exposure time might be an important factor.

We refute the second hypothesis that the sensibility to chitosan would be similar in all strains; here, we showed that the EC_50_ varied from 0.47 to >8 mg chitosan L^−1^. Mucci et al. [46] also found an intraspecific variability (EC_50_ of 0.41 and >8 mg chitosan L^−1^) between two strains of *Planktothrix agardhii* equal to what we observed here for *M. aeruginosa*. All the strains tested in this study were uni-and bicellular, which implies that the among strain variability seems to be caused by strain specific characteristics rather than a colonial or unicellular appearance. In addition, the presence or absence of MCs is not related to chitosan sensitivity, as both the MC-producing wild-type PCC 7806 and its MC-lacking mutant PCC 7806 ∆mcyB were equally sensitive.

The among strain variability can be explained by differences in the composition of the outer layer and amount/composition of extracellular polysaccharides (EPS) [58]. EPS are mainly composed of polysaccharides and proteins [59]. Due to a large number of negatively charged functional groups, their efficiency in removing heavy metals and organic contaminants protects the cells [60,61,62,63,64]. EPS also protects *M. aeruginosa* against strong oxidizers like hydrogen peroxide [60], and consequently, follow-up studies could explore the role of EPS in among strain variability and among species variability in sensitivity to chitosan. Another factor that might play a role in chitosan’s sensibility is the charge density of the membrane in each species/strain. Positively charged chitosan will have electrostatic interactions with the negatively charged cell wall of the cyanobacteria, where a higher negative charge density will lead to a stronger interaction with chitosan [65]. Stronger interactions can cause membrane destabilization and disruption of the membrane, leading to leakage of intracellular substances [29], as observed in our study.

A literature research on the use of chitosan to flocculate cyanobacterial revealed that about half of the studies did not analyse any cell health aspects (Table A2). Considering chitosan as a tool to be applied in water bodies to remove cyanobacteria, it is important not only to look at removal/coagulation efficiency but also on possible side effects on the aquatic community. From the studies that included a cell viability indicator, one third showed a negative effect of chitosan on the cells, and two-thirds did not report any adverse effect (Table A2).

In analogy with our expectation that *M. aeruginosa* would only be affected at the highest chitosan dose, we hypothesized that only at these exposures, microcystins would be released from the cells. Our results, however, are not in line with this third hypothesis. Extracellular microcystins (MCs) could be detected in the filtrates from all the strains tested, albeit not always at levels allowing quantification. Nonetheless, in strain PCC 7820 already at 2 mg chitosan L^−1^ MC release was significantly higher than in the controls. In the strain PCC 7806, extracellular MC concentration was elevated at 4 mg L^−1^ and increased at 8 mg chitosan L^−1^. Since the MC concentrations in these treatments were high, such chitosan doses should be used with care when used to treat blooms in drinking water supplies. The chitosan capacity to remove extracellular MCs has been reported and can be substantial (e.g., [47,66]). In addition, Miranda et al. [41] using field samples dominated by *M. aeruginosa* showed that exposure for two hours to chitosan significantly lowered extracellular MC concentration. The positively charged chitosan molecules probably interact with negatively charged microcystins that have a -1 charge over a broad pH range [67]. In our study, however, a clearly different chitosan effect was observed, namely the release of intracellular MCs. It is likely that extracellular MCs were first reduced, but evidently the chitosan-induced cell leakage led to significantly enhanced extracellular MC concentrations compared to non-exposed cells. Likewise, when Miranda et al. [41] used water dominated by the sensitive cyanobacterium *Cylindrospermopsis raciborskii*, they found not only strongly reduced PSII efficiency but also enhanced extracellular saxitoxins. Hence, differences in sensitivity of *Microcystis* used, exposure duration too short to evoke cell lysis (e.g., [41,66]) or matrix effects on the MC detection could underlie the apparent differences. Pei et al. [47] used an ELISA kit to measure MCs, but possible matrix effects on the antibodies were not determined. Our study showed that chitosan did not interfere with our LC-MS/MS method for MC analysis.

Studies that combined chitosan and a ballast compound revealed a reduction in extracellular MCs. For instance, a mesocosm experiment performed by Pan et al. [49] showed that chitosan-modified soil (MLS) decrease dissolved MC. Similarly, Li and Pan [35] found a reduction in MC when MLS was applied, however, when only chitosan was added, an increase in MC was observed, indicating that the decreased MC concentrations might be related to the soil MC adsorption capacity instead of to chitosan. Miranda et al. [41] found that extracellular MC concentrations were significantly reduced in treatments where chitosan was combined with a ballast compared to in chitosan only treatments. In contrast, they found higher extracellular saxitoxin concentrations when *C. raciborskii* was exposed either to chitosan alone or chitosan combined with soils and clay. While electrostatic interactions of chitosan with MCs can be expected, this is less likely for positively charged saxitoxins [68]. The study of Miranda et al. [41] underpins that when chitosan is to be applied in drinking water reservoirs, depending on the cyanobacteria prevailing, corresponding cyanotoxin analysis is strongly advised.

Any material used to mitigate cyanobacterial nuisance that causes the release of toxins is a double-edged sword. On one side, if the nuisance is reduced, this will be viewed as positive, but if cells are killed rapidly and toxins released, the water body might not be suitable for drinking, irrigation, or recreational purposes [69]. However, when such cell death happens later and close to the sediment, released toxins can be degraded (e.g., [35]) with far less impact on ecosystem functionality. Thus, the use of ballast together with chitosan (a “floc and sink” approach or the MLS technique) seems a better strategy than using only chitosan, not only because it might prevent higher concentrations of toxins in the water column but also because a ballast prevents cell accumulation at the water surface. Recently, it has been shown that damaging the cells first with hydrogen peroxide before adding the coagulant and the ballast (kill, Floc & Sink) could be a promising approach to keep *P. rubescens* precipitated [70]; in this case, chitosan could also be an alternative if used together with a ballast. 

The increase in total Chlorophyll-*a* (Figure A2) does not reflect an increase in biomass but is a result of pigments leaking out of the cells, which was confirmed by the increase in extracellular Chlorophyll-*a* (Figure 2). A rapid increase in extracellular Chlorophyll-*a* is a strong indicator of cell lysis, as is a rapid increase in extracellular MC concentration. Cell lysis implies membrane damage, which was confirmed by the membrane viability assay, wherein for all the strains tested, a green fluorescence was observed at the highest chitosan concentration used (for example Figure 4, Figure A5 and Figure A6). Sytox Green has a high affinity with nucleic acids, however, it is not able to penetrate living cells. Yet, when the membrane integrity is compromised, the stain can colour the genetic material with a bright green colour [71], as observed here in the chitosan treatments. The absence of green colour in the control, but the presence of natural red fluorescence of cyanobacteria, indicates no membrane damage in the controls (Figure 4). In the strains in which it was possible to quantify MCs, a significant positive linear relation between MC concentration and extracellular Chlorophyll-*a* was observed (*r*^2^ = 0.98 *p* < 0.0001 for PCC7820 and *r*^2^ = 0.99 *p* < 0.0001 for PCC 7806) (Figure 2). Hence, when dissolved toxins analysis is not possible, extracellular Chlorophyll-*a* might be used as a surrogate to give insight into possible toxin release.

The strain CYA 140 was the second most sensitive strain (Table 1), yet the extracellular Chlorophyll-*a* in the chitosan treatments was not as high as for other strains such as PCC 7820 and PCC 7806, which showed higher EC_50_. The absence of high concentrations of extracellular Chlorophyll-*a* for CYA 140 could be related to quick degradation of Chlorophyll-*a*. It is well known that dissolved chlorophyll-a is extremely unstable, and it will be degraded when exposed to light. It could also be related to a slower membrane damage, so the cells are not physiologically well, thus EC_50_ is low but intercellular contents are released slower. Clearly, more research is needed to decipher the cause of the observed differences between strains.

Geo-engineering materials used to manage eutrophication and control cyanobacterial blooms must be efficient, easy to apply, cheap and safe, which means it is important to be aware of all the consequences that any material can cause in the ecosystem [50]. Here, Chitosan was able to damage *M. aeruginosa* cells causing cell lysis and consequently microcystin and pigment release. These effects were, however, strain dependent. Evidently, these trials need a follow up with natural seston dominated by *M. aeruginosa*, which in the field is usually found in its typical colonial form contrasting the unicellular morphology in laboratory cultures [72]. Considering the high diversity of cyanobacteria when chitosan is considered to be used in lake restoration, the best approach to understand its effects is to test it directly on the natural biota being targeted. Such tests should include not only coagulation efficiency but also cell viability. We highlighted the importance of controlled experiments to understand the implications and efficiency of materials used to mitigate cyanobacterial nuisance. Such tests are the first step, and to predict real effects a tiered approach from laboratory to field tests is needed.

## 4. Materials and Methods

### 4.1. Microcystis aeruginosa Cultures

The eight different strains used in the experiments were obtained from different culture collections (Table 2) and were cultivated on modified WC medium [73] under controlled conditions at 22 °C with a 16:8 h light–dark cycle and 45 µmol quanta m^−2^ s^−1^ light intensity. Before the experiment, the cultures were refreshed twice (around two weeks interval), always in the exponential phase.

### 4.2. Chitosan

Chitosan was obtained from Polymar Ciência e Nutrição S/A, and the deacetylation degree was 86.3% (Batch-010913, Fortaleza, CE, Brazil), and there is no information on the molecular weight. The chitosan (made of shrimp shells) was acidified with 96% acetic acid solution (Merck, analytical grade, VWR International B.V., Amsterdam, The Netherlands), yielding a final concentration of 0.1% acetic acid. 

### 4.3. Experiment Design

Aliquots of *M. aeruginosa* were transferred to 100 mL Erlenmeyer containing 50 mL of modified WC medium, yielding a final concentration of 100 µg Chlorophyll-*a* L^−1^. Six concentrations of Chitosan were used (0, 0.5, 1, 2, 4 and 8 mg L^−1^) based on the concentrations frequently used to flocculate cyanobacteria in lake restoration [26,35,37,40,45]. The experiment was done in triplicate. An extra control was added in which only acetic acid was added in the same dose as in chitosan treatment to check if the acetic acid in which chitosan was dissolved had any influence on *M. aeruginosa* cells. After the addition of chitosan or acid acetic, the flasks were mixed and placed in the laboratory at 22 °C in 16:8 h light–dark cycle at 45 µmol quanta m^−2^ s^−1^. After 1, 4, and 24 h, subsamples were taken to measure the total Chlorophyll-*a* concentration and Photosystem efficiency II (PSII) through PHYTOPAM phytoplankton analyser (Heinz WalzGmbH, Effeltrich, Germany). Additionally, at the end of the experiment, 3 mL samples from each flask were filtered through a filter unit (Aqua 30/0.45CA, Whatman^®^, VWR International B.V., Amsterdam, The Netherlands) and measured again in the PHYTOPAM to quantify Chlorophyll-*a* released from the cells. After 24 h, pH was measured in each flask, and 8 mL samples were filtered through glass fiber filters (GF/C, Whatman^®^, VWR International B.V., Amsterdam, The Netherlands) and placed in glass tubes for dissolved microcystin (MC) analysis. The samples were dried in a Speedvac concentrator (Savant^TM^ SPD121P, Thermo Fisher Scientific, Asheville, NC, USA) and were reconstituted in 900 µl methanol (J.T. Baker^®^, 97%, VWR International B.V., Amsterdam, The Netherlands). After that, the reconstituted samples were transferred to a 1.5 mL tube with a cellulose-acetate filter and centrifuged for 5 min at 16,000× *g*. The filtrates were transferred to amber glass vials and analysed for eight MC variants (MC–dmRR, RR, YR, dmLR, LR, LY, LW, and LF) using LC-MS/MS according to Lürling and Faassen [6]. The MC analysis was performed for the strains MiRF-1, PCC 7806, PCC 7820, and CYA 140 (Table 2).

### 4.4. Cell Membrane Permeability

An aliquot from each replica was taken, joined, and centrifuged at 5000× *g* for 10 min to evaluate chitosan’s effect on membrane integrity, immediately after 24 h of exposure. The pellet was stained with Sytox^®^ Green (Thermo Fisher Scientific, Waltham, MA, USA) at a final concentration of 1nM for 30 min in the dark. The samples were observed under a fluorescence microscope (ZEISS, Axioimager D2, Jena, Germany) using the filter long pass for Fluorescein (450–490 for excitation and 515 nm for emission). Sytox^®^ Green binds to nucleic acid, but it cannot penetrate the cell membrane. However, a damaged membrane allows the stain to infiltrate, resulting in a green fluorescence colour when analysed in a fluorescence microscope.

### 4.5. Matrix Effect on MC Analysis

The possible effect of chitosan on the toxins analysis was evaluated by incubating pure microcystin mix standards (all eight variants: MC–dmRR, RR, YR, dmLR, LR, LY, LW, and LF) for 24 h in a solution with 8 mg Chitosan L^−1^ dissolved in WC medium. The control series contained only WC medium and pure microcystin mix standards. The test was performed using three replicas, and MC analysis was executed as mentioned before.

### 4.6. Data Analysis

The PSII for each strain at each time point was compared between different chitosan concentrations using one-way ANOVA or Kruskal–Wallis One Way Analysis of Variance on Ranks when the normality test (Shapiro–Wilk) or Equal Variance test (Brown–Forsythe) failed. For each strain, the chitosan concentrations that caused a 50% reduction in their PSII efficiency compared to the control (EC_50_) were determined by non-linear regression using four logistic parameter curves in the software Sigma Plot 13.0. EC_50_ values were statistically compared between strains using one-way ANOVA. Extracellular MC and filterable Chlorophyll-*a* concentration were compared between different chitosan concentrations using the one-way ANOVA or Kruskal–Wallis One Way Analysis of Variance on Ranks when the normality test (Shapiro–Wilk) failed. The effect of chitosan on MC standards was tested by Student’s *t*-test.

## Figures and Tables

**Table 1:** Mean EC_50_ values (mg L^−1^; with standard deviation, *n* = 3) of chitosan for the Photosystem II efficiency in different *M. aeruginosa* strains. Letters (A, B and C) represent homogenous groups (Tukey pairwise comparisons).

*M. aeruginosa* Strain	EC_50_-24 h (mg L^−1^)
MiRF-1	>8
PCC 7806 ΔmcyB	>8
PCC 7806	>8
PCC 7820	4.51 (0.37) *p* < 0.0001^A^
PCC 7005	3.44 (0.42) *p* < 0.0001^B^
SAG 14.85	1.71 (0.08) *p* < 0.0001^C^
CYA 140	1.06 (0.04) *p* < 0.0001^C^
SAG 17.85	0.47 (0.05) *p* < 0.0001^C^

**Table 2:** *Microcystis aeruginosa* strains used in the experiments and the microcystin (MC) variants that have been found in them.

Strain ID	Acquired from	Microcystins (MCs) Produced
MiRF-1	Laboratory of Ecophysiology and Toxicology of Cyanobacteria (Brazil)	dm-MC-LR, MC-LR, MC-LY, MC- LW, MC-LF [74]
PCC 7806 ΔmcyB	Pasteur Culture Collection (France)	None [75]
PCC 7806	Pasteur Culture Collection (France)	dm-MC-LR, MC-LR [76]
PCC 7005	Pasteur Culture Collection (France)	None detected (this study)
PCC 7820	Pasteur Culture Collection (France)	dm-MC-LR, MC-LR, MC-LY, MC-LW, MC-LF [77]
SAG 14.85	Sammlung von Algenkulturen der Universität Göttingen (Germany)	dm-MC-LR, MC-LR (unpublished data)
SAG 17.85	Sammlung von Algenkulturen der Universität Göttingen (Germany)	dm-MC-LR, MC-LR, MC-YR [76]
CYA 140	Norwegian Institute for Water Research (Norway)	dm-MC-LR, MC-LR [76]

## Appendix A

**Table A1:** F- and *p*-values of one-way ANOVAs and H- and *p*-values of Kruskal–Wallis One Way Analysis of Variance on Ranks when normality tests failed (Shapiro–Wilk) for Photosystem II efficiencies in eight different *M. aeruginosa* strain exposed for 1, 4 and 24 h to six different concentrations chitosan (0 to 8 mg L^−1^).

*M. aeruginosa* Strain	Exposure Duration		
	1 h	4 h	24 h
MiRF-1	H_5_ = 9.93; *p* = 0.077	F_5,12_ = 4.65; *p* = 0.014 *	F_5,12_ = 13.87; *p* < 0.001 *
PCC 7806 ΔmcyB	H_5_ = 16.70; *p* = 0.005 *	F_5,12_ = 66.34; *p* < 0.001 *	H_5_ = 12.70; *p* = 0.026 *
PCC 7806	H_5_ = 12.24; *p* = 0.032 *	H_5_ = 11.28; *p* = 0.046 *	H_5_ = 15.81; *p* = 0.007 *
PCC 7820	F_5,12_ = 89.62; *p* < 0.001 *	F_5,12_ = 47.06; *p* < 0.001 *	F_5,12_ = 1079.0 *p* < 0.001 *
SAG 14.85	H_5_ = 16.317; *p* = 0.006 *	F_5,12_ = 876.16; *p* < 0.001 *	H_5_ = 16.1; *p* = 0.007 *
CYA 140	F_5,12_ = 149.94; *p* < 0.001 *	F_5,12_ = 144.96; *p* < 0.001 *	F_5,12_ = 338.1; *p* < 0.001 *
PCC 7005	H_5_ = 14.13; *p* = 0.015 *	F_5,12_ = 187.49; *p* < 0.001 *	H_5_ = 15.16; *p* = 0.01 *
SAG 17.85	H_5_ = 16.74; *p* = 0.005 *	H_5_ = 16.74; *p* = 0.005 *	H_5_ = 16.56; *p* = 0.005 *

* represents the significant statistical differences.

**Table A2:** Scientific papers that studied chitosan coagulation efficiency or/and the effect on cyanobacteria/dinoflagellates.

Literature Available	Endpoints	Effect
[46]	PSII efficiency and Membrane permeability (MP)	−
[47]	K^+^ release and dissolved toxins	−
[78]	Phycocyanin and allophycocyanin release	−
[48]	K^+^/M^2+^ release and dissolved toxins	−
**This study**	PSII efficiency, dissolved toxins, MP and FChl-*a*	−
[40]	PSII efficiency	0
[79]	PSII efficiency	0
[35]	Dissolved toxins	0
[21]	Cell viability and recovery	0
[55]	PSII efficiency	0
[66]	K^+^ release	0
[80]	Growth	0
[45]	PSII efficiency	0
[42]	PSII efficiency	0
[41]	PSII efficiency and dissolved toxins	0 and −
[43]	Dissolved toxins	0 and −
[81]	NR	NR
[82]	NR	NR
[83]	NR	NR
[84]	NR	NR
[35]	NR	NR
[85]	NR	NR
[86]	NR	NR
[38]	NR	NR
[87]	NR	NR
[88]	NR	NR
[37]	NR	NR
[36]	NR	NR
[25]	NR	NR
[26]	NR	NR
[89]	NR	NR

− means negative effect on the biota target, 0 means no negative effect observed and NR means no effect reported.
